# Reduced Glomerular Function and Prevalence of Gout: NHANES 2009–10

**DOI:** 10.1371/journal.pone.0050046

**Published:** 2012-11-27

**Authors:** Eswar Krishnan

**Affiliations:** Department of Medicine, Stanford University School of Medicine, Palo Alto, California, United States of America; Harvard Medical School, United States of America

## Abstract

**Background:**

The renal tubule is a major route of clearance of uric acid, a product of purine metabolism. The links between reduced glomerular filtration rate (GFR), hyperuricemia, and gout in the general population are not well understood. The objective of the present study was to estimate prevalence of gout and hyperuricemia among people with impaired GFR in the US general population.

**Study Design:**

Cross-sectional, survey-weighted analyses of data on adults (age>20 years) in the 2009–10 cycle of the US National Health and Nutrition Examination Surveys (n = 5,589). Associations between self-reported physician diagnosis of gout and degrees of renal impairment were the primary focus of the present analyses.

**Results:**

In the 2009–2010 period, there was an estimated 7.5 million people with gout in the US. There were 1.25 million men and 0.78 million women with moderate or severe renal impairment and gout. The age standardized prevalence of gout was 2.9% among those with normal GFR compared to 24% among those with GFR<60 ml/min/1.73 m^2^.In multivariable logistic regression analyses that adjusted for age, gender, body mass index, hypertension, diabetes, hypertension medications, including diuretics, blood lead levels, and hyperlipidemia, the odds ratios of gout and hyperuricemia were 5.9 (2.2, 15.7) and 9.58 (4.3, 22.0) respectively among those with severe renal impairment compared to those with no renal impairment. Approximately 2–3 fold increase in prevalence of gout was observed for each 30 ml/min/1.73 m^2^ decrease in GFR, after accounting for the above factors.

**Conclusions:**

Renal glomerular function is an important risk factor for gout**.** The prevalence of hyperuricemia and gout increases with decreasing glomerular function independent of other factors. This association is non-linear and an eGFR of 60 ml/min/1.73 m^2^ appears to be a threshold for the dramatic increase in the prevalence of gout.

## Introduction

Worldwide, chronic kidney disease (CKD) is a growing public health problem; in the United States, CKD affects approximately 1 in 6 adults. [1], [2], [3] The prevalence of CKD is higher in older age and in males, and is lower in Hispanics than in other ethnicities. [4] Epidemiological studies suggest that the prevalence of CKD may be increasing, in part due to an increase in the prevalence of risk factors such as diabetes, hypertension, and obesity. [3].

The kidneys play an important role in the excretion of urate, a by-product of purine metabolism. Although almost all serum urate is freely filtered in the glomeruli, a large proportion is reabsorbed and then actively secreted. [5] The decrease in glomerular filtration rate that occurs in renal impairment results in an adaptive *increase* in fractional excretion of urate and urate excretion per nephron. [6], [7] In severe renal impairment (inulin clearance <15 ml/min), glomerular filtration becomes the rate limiting step and up to 45% of the filtered urate is excreted. [8] Additionally, there is an increase in extra-renal clearance of urate through the liver and the gut, although this mechanism may be overwhelmed in severe renal impairment. [7].

Despite the pathophysiological links, there have been relatively few studies that have examined the prevalence of gout among patients with renal impairment. Two large studies from France in the 1960s and 1980s found that the prevalence of gout among those with renal impairment was no more than 1%. [9], [10] Another large study from the US in 1975 reported that out of the 1700 patients with gout studied, only 84 (4.9%) had primary renal disease that preceded the onset of gout. [11] More recently, data from cohort of 18,358 diabetics in New Zealand suggested that declines in renal function were associated with increased risk for gout. [12] Cross-sectional analyses of data from general practice registers in the United Kingdom suggested that having a diagnosis of chronic renal failure is associated with a 2.5-fold higher likelihood of concomitant diagnosis of gout. [13], [14], [15] Due to underdiagnosis in general clinical practice, these studies may have underestimated the magnitude of the gout risk. [16], [17] In spite of these associations, the prevalences of gout and hyperuricemia among those with renal impairment in the general population have not been reported.

Although renal impairment is present in over 40% of patients with gout, there are few safe, effective, and regulatory body-approved treatments for hyperuricemia of gout for patients with moderate to severe renal impairment. [18], [19] The main objective of this study was to document the prevalence rates and the burden of illness of gout among those with renal impairment in the contemporary United States.

## Methods

### Data and Design

Post-hoc analysis of data from the latest cycle of a nationally representative cross sectional survey in the US, the National Health and Nutrition Examination Surveys (NHANES, 2009–2010), was performed. All participants provided informed consent for data collection and for the data to be publicly disseminated in a de-identified format. This study is exempt from formal Ethics committee approval, as it involves de-identified data freely available over the internet (http://www.cdc.gov/nchs/nhanes.htm, accessed September 20, 2012).

### Data Collection

The sampling frame of participants in the NHANES is all the non-institutionalized adults and children in the US population. An exhaustive description of the survey design, data collection strategies and instruments is available online. [20] In brief, this survey is a complex multistage sample of the US population, where the basic geographic unit is the county. The survey deliberately oversamples patient subgroups that are difficult to enroll. The survey had three major data collection components: (*i*) a telephone interview, (*ii*) an in-person study visit with additional questionnaires, anthropometry and other biometric measurements, and (*iii*) laboratory testing including a fasting phlebotomy. Self-reported data on use of diabetes and hypertension medication were available, although specific medication data were not available for the present analyses.

### Inclusion and Exclusion Criteria

All adults (age >20) who completed a household interview and laboratory visit and who were not pregnant or breast feeding were included. A very small number of participants (<22) self-reported receiving dialysis. This group was not excluded.

### Laboratory Testing

Fasting serum specimens were processed, stored, and shipped to the Collaborative Laboratory Services for analysis. Detailed specimen collection, assays, standardization, calibration, and processing protocols are described in the NHANES Laboratory/Medical Technologists Procedures Manual. [21] Serum creatinine was assayed using the Jaffe rate method, and urate was assayed by the uricase method.

### Case Definitions and Calculated Variables

The standard NHANES case definition for gout was used for the present analyses. [22] All participants completing the medical history questionnaire were informed that “Gout is one of the most painful forms of arthritis. It occurs when too much uric acid builds up in the body. For many people, the first attack of gout occurs in the big toe. Often, the attack wakes a person from sleep.” Subsequently, they were asked, “Has a doctor or health professional ever told you that you have gout?” Those who responded yes were assessed as having gout. Methodology similar to this has been validated and found reliable in large population-based studies. [23], [24].

Hyperuricemia was defined as a serum urate level of >7.0 mg/dL among men and >6 mg/dL for women, similar to the definition used in other studies. [25], [26].

Estimated glomerular filtration rate (eGFR) was calculated using the CKD-EPI creatinine equation, as previously described. [27] Urinalysis results were not available for the participants in the survey. Based on eGFR, we categorized participants as normal (≥90 ml/min/1.73 m^2^) or as having mild renal impairment (60–89 ml/min/1.73 m^2^), moderate renal impairment (30–59 ml/min/1.73 m^2^) or severe renal impairment (<30 ml/min/1.73 m^2^). For analyses where renal impairment was dichotomized, we classified subjects with eGFR greater than or equal to 90 ml/min/1.73 m^2^ as normal and the rest as suffering from renal impairment.

Hypertension was defined as a mean blood pressure of ≥140 mm Hg or a diastolic blood pressure of ≥90 mm Hg. [28] Current use of antihypertensive drugs categorized the individual as hypertensive, regardless of the actual blood pressure measurement. Diabetes was defined as a fasting glucose concentration of 126 or greater or current use of anti-diabetic medications. [29] Metabolic syndrome was defined according to the ATP guidelines described by Grundy *et al*. [30] In patients whose waist circumferences were not available, we considered a body mass index of ≥30 kg/square meter as meeting the waist circumference criterion for metabolic syndrome. Hyperlipidemia was defined as the presence of one or more of the following serum measures: total cholesterol>200 mg/dL; triglycerides >200; high density lipoproteins <40 mg/dL; low density lipoproteins >130 mg/dL. Current use of cholesterol lowering medications classified an individual as hyperlipidemic. Alcohol consumption was assessed using the self-reported number of days in the prior month when the participants drank alcohol. Blood lead concentrations, known to be associated with gout, were measured by inductively coupled plasma mass spectrometry, using standard NHANES protocol. [31], [32], [33] This measure was log-transformed because the distribution was skewed.

Ethnicity information was self-identified. There were too few participants in the categories outside whites, African Americans, and Hispanics to be analyzed individually. Among Hispanics, we combined those of Mexican and non-Mexican origins into a single Hispanic category. Financial status of the participant was assessed using the poverty income ratio, the ratio of a family’s income to the US Census Bureau’s poverty threshold, which varies with the number and ages of family members and is revised yearly. [34].

### Statistical Analyses

All analyses were performed as per the NHANES guidelines. [35] Unless specified otherwise, all analyses were performed using the survey suite of commands in STATA 11 (SVY, StataCorp, and College Station, TX). These analyses incorporated the study visit weights, primary sampling unit, and stratification design of the study, enabling estimation of the number of people in the US with gout. Rates were calculated as the proportion of participants with gout in each category. Age standardization was performed with the year 2000 census standard. [36].

The relationship between hyperuricemia, gout and renal function was analyzed using survey weighted logistic regressions. Serum urate level was also analyzed as a continuous measure in ordinary least squared regressions. In these models the key dependent variables were gout and hyperuricemia/serum urate. The key independent variable was renal impairment. The adjusting variables were age, gender, body mass index, diabetes (present/absent), hypertension (present/absent), poverty ratio, ethnicity (Whites, African Americans, Hispanics, and others), alcohol consumption (quartiles), hyperlipidemia (present/absent), and current use of blood pressure medications (present/absent). All these variables were clinically meaningful and were included in the multivariable models regardless of the bivariate analysis results. Preplanned subgroup analyses were performed based on clinical rationale for selected variables, such as ethnicity and metabolic syndrome.

## Results

Overall, there were 10,537 observations in the NHANES 2009–10, from which we excluded 4,319 participants less than 20 years of age, 99 women who were pregnant or breastfeeding and 530 observations due to missing values of gout, hyperuricemia or serum creatinine, leaving 5,589 observations in the analysis dataset. The 99^th^ percentile of serum creatinine measured was 1.86 mg/dL (164 µmol/L); 22 participants in the dataset reported receiving hemodialysis or peritoneal dialysis in the preceding 12 months. **Table** **1** shows the characteristics of the study sample by renal impairment status.

**Table 1:** Comparison of participants by renal function.^a^

		Severity of Renal Impairment			
	None	Mild	Moderate	Severe	Overall
**Age, years**	34 (16)	60 (15)	73 (9)	69 (15)	44 (21)
**Men, %**	48%	53%	49%	47%	49%
**Whites, %**	38%	61%	64%	55%	46%
**Hispanics, %**	36%	20%	14%	17%	31%
**African Americans, %**	19%	14%	18%	20%	18%
**Others, %**	6%	5%	3%	8%	6%
**Poverty ratio, (range 0–5)**	2.21 (1.59)	2.76 (1.66)	2.46 (1.48)	2.18 (1.41)	2.37 (1.61)
**Hypertension, %**	17%	51%	80%	88%	31%
**Current blood pressure medication, %**	12%	39%	69%	84%	25%
**Diabetes mellitus, %**	7%	15%	28%	33%	10%
**Current diabetes medications, %**	1%	3%	9%	22%	3%
**Hyperlipidemia, %**	51%	73%	74%	73%	58%
**Metabolic syndrome, %**	13%	23%	35%	38%	17%
**Body mass index, kg/m^2^**	28 (7)	29 (6)	30 (7)	29 (6)	28 (7)
**Waist circumference, cm**	94 (17)	101 (15)	104 (15)	102 (16)	96 (17)
**Systolic blood pressure, mm Hg**	116 (16)	127 (19)	133 (21)	133 (25)	120 (18)
**Diastolic blood pressure, mm Hg**	67 (4)	70 (13)	62 (14)	60 (17)	67 (14)
**Log (blood lead level in microgram/dL)**	0.01 (0.67)	0.44 (0.58)	0.6 (0.54)	1.01 (0.73)	0.17 (0.68)
**Serum urate, mg/dL**	5.12 (1.33)	5.71 (1.39)	6.74 (1.54)	6.94 (2.09)	5.4 (1.44)
**Serum creatinine, mg/dL**	0.76 (0.16)	0.96 (0.17)	1.3 (0.25)	3.86 (2.78)	0.88 (0.45)

a Data presented are unweighted means (standard deviation) unless otherwise specified. Kidney disease classified based on estimated glomerular filtration rates (normal > = 90, mild 60–89, moderate 30–59, severe <30 mL/min/1.73 m^2^). Gout was defined as self-reported physician/provider diagnosis. See methods section for details. Diabetes was defined as fasting glucose >125 or use of anti-diabetic medications. Hypertension was defined as systolic blood pressure >140 mmHg, diastolic blood pressure >90 mmHg or use of blood pressure medications. Blood pressure medications included diuretics. Poverty ratio was the ratio of household income to the Federal poverty levels.

**Table 2** shows the estimated number of gout cases in the US by renal function, age, gender, and ethnicity.

**Table 2:** Estimates of number of people with gout and hyperuricemia, in millions, by renal impairment status.^a^

	Gout^b^				Hyperuricemia^c^			
	Severity of Renal Impairment				Severity of Renal Impairment			
	None	Mild	Moderate	Severe	None	Mild	Moderate	Severe
**Overall**	1.72	3.83	1.61	0.42	16	15	6	1
**Age**								
** 20–40**	0.44	0.14	<0.1	<0.1	8.37	2.44	<0.1	<0.1
** 40–65**	1.16	1.49	0.26	<0.1	7.40	7.60	1.50	<0.1
** 65+**	0.12	2.19	1.27	0.38	0.30	4.60	4.80	0.78
**Gender**								
** Men**	1.52	2.45	1.01	0.24	11.10	8.80	2.20	0.20
** Women**	0.20	1.38	0.60	0.18	4.90	5.90	1.20	0.68
**Ethnicity**								
** Whites**	1.30	3.20	1.30	0.33	1.03	11.00	5.02	0.65
** Hispanics**	<0.1	0.10	<0.1	<0.1	2.20	1.10	0.33	<0.1
** African Americans**	0.26	0.41	0.15	<0.1	2.21	1.54	0.77	0.13
** Others**	0.10	<0.1	0.11	<0.1	1.40	0.93	0.29	<0.1

a Kidney disease classified based on estimated glomerular filtration rates (normal > = 90; mild 60–89; moderate 30–59 severe <30 mL/min/1.73 m^2^).

b Gout was defined as self-reported by physician/provider diagnosis. See methods section for details.

c Hyperuricemia was defined as serum urate >7.0 mg/dL (>416 micromoles/L) for men and >6.0 mg/dL (>357 micromoles/L).

**Figure 1:**
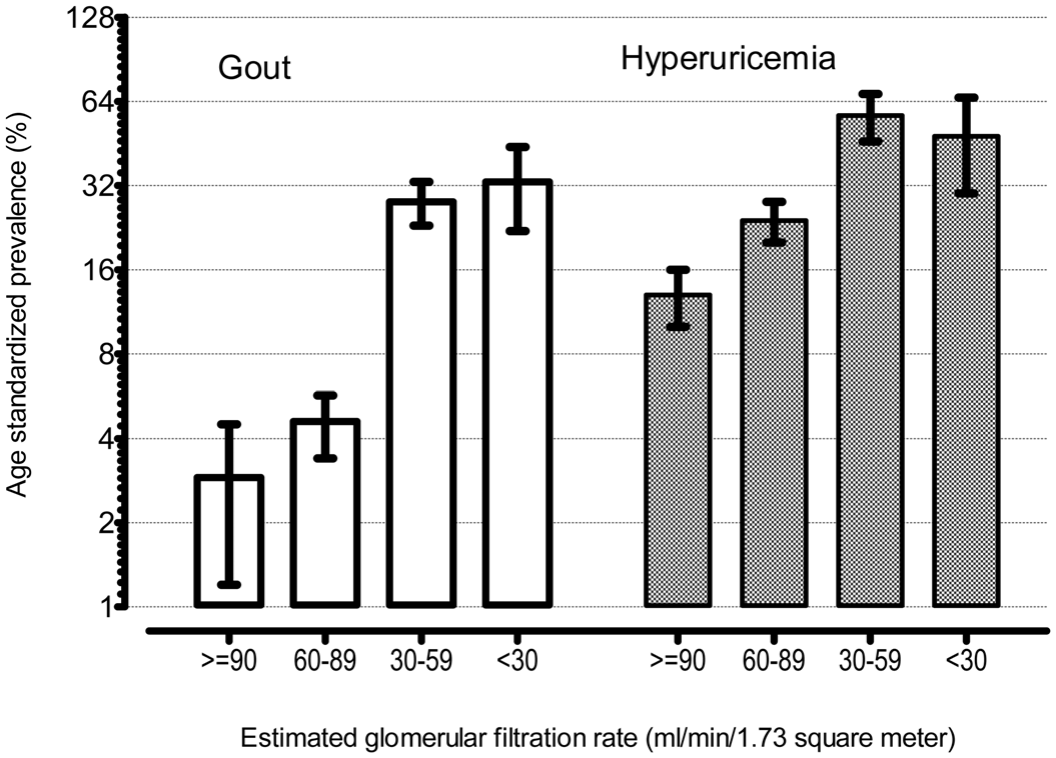
Age-standardized prevalence rates of gout and hyperuricemia. Gout was defined as self-reported physician/provider diagnosis. Hyperuricemia was defined as serum urate >7.0 mg/dL for men and >6 mg/dL for women. Trend tests performed by survey weighted logistic regressions where age and estimated glomerular filtration rates were utilized as continuous measures were statistically significant in both the cases (p<0.001). Conversion factors for units: serum urate in mg/dL to µmole/L, × 59.48.

**Figure 2:**
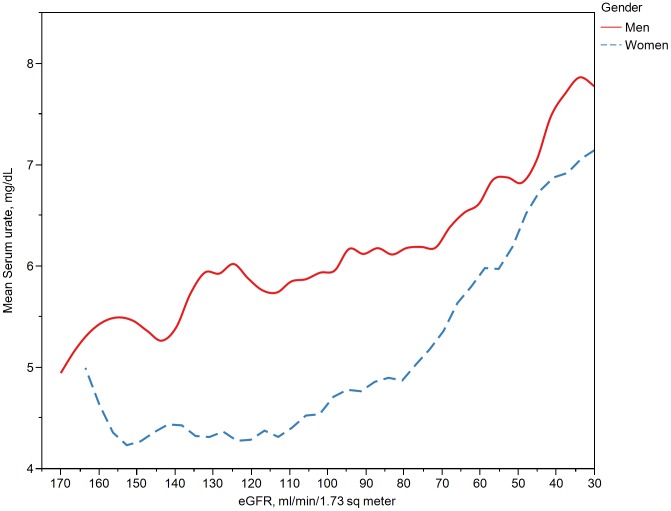
Bivariate association between estimated glomerular filtration rate and serum urate concentrations. The curves based on mean serum urate concentration vs. eGFR were fitted by locally weighted scatterplot smoothing (lowess) regressions using unweighted data. Conversion factors for units: serum urate in mg/dL to µmole/L, × 59.48.

Overall, the unadjusted prevalence of gout among those with any renal impairment was five-fold higher than among those without any renal impairment [7.3%; (6.0%, 8.8%) vs. 1.4% (0.9%, 2.1%)]. The unadjusted rates among men with and without any renal impairment were 9.3% (6.8%, 12.5%) and 2.5% (1.6%, 3.9%), respectively. Among women, the corresponding rates were 5.3% (3.8%, 7.2%) and 0.3% (0.1%, 0.8%). **Table** **3** shows the estimated crude and age-adjusted prevalence of gout, hyperuricemia, and categories of renal impairment that were comparable to the estimates from the 2007–8 NHANES cycle. The present analyses estimate that there were 7.58 million with gout in the US in 2009–10. Among these, there were 5.86 million people with eGFR <90 ml/min/1.73 m^2^. **Figure 1** shows the increasing prevalences of gout and hyperuricemia with worsening renal function. The age standardized prevalence of gout was 2.9% (1.6%, 5.1%) among those with no renal impairment compared to 24.0% (20.3%, 28.2%) among those with moderate or severe renal impairment. Similarly there was a five-fold increase in prevalence of hyperuricemia among those with severe renal impairment compared to those with no renal impairment. **Figure** **2** shows the increase in mean serum urate with decrease in renal function among men and women. Urate concentrations among men were higher in the subset of the population with higher eGFR but the gender differences disappeared at low eGFR (<30 ml/min/1.73 m^2^).

**Table 3:** Overall prevalence of gout, hyperuricemia and renal impairment in the NHANES 2009–10.^a^

	Number of observationsin the dataset	Population Estimatemillions	Unadjusted Prevalence,% (95% confidence interval)	Age Standardized Prevalence,% (95% confidence interval)
**Gout**	277	8.1	3.8 (3.0, 4.7)	4.3 (3.6, 5.2)
**Hyperuricemia**	1171	37.9	18.7 (17.1, 20.3)	19.3 (17.8, 20.9)
**Renal Impairment**				
** None**	4543	122.7	60.3 (57.2, 63.3)	56.1 (54.4, 57.7)
** Mild**	1804	67.1	33.0 (30.5, 35.5)	36.5 (34.7, 38.4))
** Moderate**	449	12.1	6.0 (5.1, 7.0)	6.7 (5.8, 7.7)
**Severe**	64	1.5	0.8 (0.5, 1.1)	0.7 (0.4, 1.3)

a No exclusions.

The prevalence rates of gout were lower in women than in men (**Table 4**). Among those with no renal impairment, Hispanics had a prevalence rate almost a tenth of Whites and African Americans. This difference prevailed in all categories of renal impairment except in the severe renal impairment category, where there were too few Hispanics with gout for a reliable estimate. Among the other ethnic categories, increasing severity of renal impairment was associated with higher prevalence rates of gout, except in those with diabetes and those with metabolic syndrome.

**Table 4:** Age-standardized prevalence (%) of gout by renal glomerular function.^a^

		Severity of Renal Impairment			
	Overall	None	Mild	Moderate	Severe
					
**Overall**	4.3 (3.6, 5.2)	2.9 (1.6, 5.1)	4.6 (3.5, 5.9)	27.9 (23.9, 32.30	33.3 (23.3, 45.2)
**Gender**					
** Men**	6.4 (5.0, 8.3)	5.7 (3.0, 10.3)	6.1 (4.6, 8.0)	31.0 (23.9, 39.2)	26.2 (14.6, 42.4)
** Women**	2.4 (1.7, 3.5)	0.7 (0.2, 1.0)	3.0 (1.7, 5.1)	7.0 (3.9, 12.2)	21.3 (17.2, 26.1)
**Ethnicity**					
** Whites**	4.5 (3.5, 5.8)	3.5 (1.5, 7.7)	4.5 (3.2, 6.3)	28.7 (23.6, 34.4)	42.9 (30.7, 56.0)
** Hispanics**	1.7 (0.8, 3.9)	0.3 (0.1, 0.5)	3.2 (1.1, 8.7)	4.3 (2.9, 6.3)	NA
** African-Americans**	5.9 (4.1, 8.4)	3.6 (2.3, 5.6)	8.0 (5.2, 11.9)	13.1 (9.6, 17.6)	32.1 (17.8, 50.7)
** Others**	2.9 (1.3, 6.2)	1.2 (0.4, 3.4)	1.7 (0.6, 4.9)	31.2 (26.5, 36.5)	25.0 (25.0, 25.0)
**Body Mass Index Kg/m2**					
** <25**	1.7 (1.0, 2.9)	1.2 (0.3, 4.3)	1.2 (0.6, 2.4)	1.4 (0.6, 3.3)	21.4 (23.5, 40.5)
** 25–30**	3.6 (2.8, 4.5)	2.1 (0.6, 6.9)	4.2 (3.1, 5.7)	5.4 (2.5, 11.2)	43.2 (29.4, 58.1)
** >30**	6.5 (5.1, 8.1)	5.3 (2.9, 9.5)	6.9 (4.6, 10.2)	31.7 (25.0, 39.3)	24.5 (18.1, 32.2)
**Hypertension** b					
** Absent**	2.5 (1.8, 3.6)	2.4 (0.5, 10.9)	2.5 (1.5, 4.1)	25.9 (20.9, 31.6)	17.7 (8.4, 33.6)
** Present**	6.1 (4.8, 7.7)	4.0 (2.3, 6.9)	6.7 (4.6, 9.5)	10.5 (6.3, 17.0)	33.2 (22.6, 45.9)
**Current anti-hypertensive therapy**					
** Absent**	2.4 (1.5, 3.5)	2.0 (0.5, 7.5)	2.4 (1.5, 4.0)	24.8 (20.9, 29.1)	12.2 (5.1, 26.1)
** present**	6.6 (5.0, 8.7)	3.9 (1.9, 8.1)	10.8 (7.6, 15.1)	11.7 (7.0, 18.9)	33.8 (23.4, 46.4)
**Diabetes mellitus** c					
** Absent**	4.0 (3.2, 5.0)	2.7 (1.5, 5.0)	4.3 (3.1, 5.9)	26.1 (23.4, 29.0)	44.1 (29.4, 59.9)
** Present**	5.8 (4.0, 8.4)	3.7 (1.6, 8.3)	6.7 (4.4, 10.1)	17.1 (9.4, 29.0)	4.2 (0.7, 21.7)
**Metabolic syndrome** d					
** Absent**	3.6 (2.9, 4.4)	2.5 (1.1, 5.2)	3.8 (2.7, 5.4)	24.5 (22.2, 26.9)	43.0 (27.5, 59.9)
** Present**	6.6 (4.7, 9.0)	4.5 (2.1, 9.3)	7.3 (4.5, 11.8)	17.4 (10.5, 27.6)	6.3 (2.1, 17.1)

a Age was standardized to US Census 2000 population. Prevalence rates given as percentage (95% confidence interval). Kidney disease classified based on estimated glomerular filtration rates (normal > = 90; mild 60–89; moderate 30–59 severe <30 mL/min/1.73 m^2^). Gout was defined as self-reported physician/provider diagnosis. Categories below 60 were combined for a more precise estimate. See methods section for details. NA: Unable to estimate due to wide variance.

b Hypertension was defined per JNC7 criteria. Current use of antihypertensive medications was deemed to indicate hypertension.

c Diabetes was defined as a fasting blood glucose >126 mg/dL or use of anti-diabetic medications.

d Metabolic syndrome was defined per ATP criteria.

Among those with gout, the age-standardized proportion of participants with normal renal function was 50% (43%, 58%) among men and 26% (16%, 38%) among women (**Table** **5**). The proportion of people with severe renal impairment among women was more than 6 times the corresponding proportion among men. Such a gender disparity was not apparent with respect to hyperuricemia.

**Table 5:** Distribution of severity of renal impairment among people with gout and hyperuricemia.^a^

	Men, % (95% confidence interval)		Women, % (95% confidence interval)	
	Unadjusted	Age-standardized	Unadjusted	Age standardized
**Participants with gout**				
No renal impairment	29 (22, 37)	50 (43, 58)	9 (3, 21)	26 (16, 38)
Mild renal impairment	47 (38, 56)	36 (29, 45)	58 (42, 73)	45 (32, 59)
Moderate renal impairment	19 (14, 26)	11 (7, 18)	25 (14, 41)	10 (5, 17)
Severe renal impairment	5 (2, 11)	3 (1, 6)	8 (3, 18)	20 (17, 24)
**Participants with hyperuricemia**				
No renal impairment	50 (44, 56)	43 (40, 47)	31 (26, 38)	50 (45, 56)
Mild renal impairment	39 (33, 46)	44 (38, 51)	38 (32, 44)	32 (27, 38)
Moderate renal impairment	10 (7, 13)	11 (8, 15)	27 (22, 33)	15 (12, 20)
Severe renal impairment	1 (0, 2)	1 (0, 3)	4 (3, 7)	2 (1, 5)

a Kidney disease classified based on estimated glomerular filtration rates (normal > = 90, mild 60–89, moderate 30–59, severe <30 mL/min/1.73 m^2^). Gout was defined as self-reported physician/provider diagnosis. See methods section for details. Hyperuricemia was defined as serum urate >7.0 mg/dL for men and >6.0 mg/dL for women. Age standardization was performed using the year 2000 US Census.

Results of logistic regression models are shown in **Table 6**. A substantial increase in the odds ratios for hyperuricemia and gout was noted with lower eGFR. Information on alcohol consumption was only available for 3,600 participants. Additional adjustment for this variable in logistic regression did not change the findings. Results of the ordinary least squares (OLS) models are shown in **Table** **7**. In multivariable OLS regressions that adjusted for the same variables as in the logistic regression models, each standard deviation decrease of eGFR (27.6 ml/min/1.73 m^2^) was associated with a 0.64 mg/dL (0.56, 0.72) increase of serum urate. Analyses including alcohol consumption information and excluding the 22 NHANES participants who reported utilizing dialysis did not alter these results.

**Table 6:** Results of logistic regression analyses of the risk for gout and hyperuricemia by renal impairment.^a^

			Severity of Renal Impairment			
	Number of observations in the model	One standarddeviation decreasein eGFR^b^	None	Mild	Moderate	Severe
**Gout**						
**Unadjusted**	5,586	3.1 (2.7, 3.6)	1	4.2 (2.6, 6.8)	10.8 (7.3, 15.9)	26.1 (10.8, 63.3)
**Adjusted for age, sex, gender** **and race**	5,586	2.1 (1.6, 3.0)	1	1.9 (1.0, 3.6)^c^	3.1 (1.5, 6.4)	7.8 (3.0, 20.8)
**Final Multivariable model**	5,360	1.8(1.3, 2.6)	1	1.8(1.0,3.2)^d^	2.4 (1.2, 4.9)	5.9 (2.2, 15.7)
**Hyperuricemia**						
**Unadjusted**	5,589	2.2 (1.9, 2.4)	1	1.9 (1.5, 2.3)	7.5 (5.7, 9.7)	9.1 (4.7, 17.8)
**Adjusted for age, sex, gender** **and race**	5,589	2.7 (2.3, 3.3)	1	2.0 (1.5, 2.5)	9.0 (6.1, 13.4)	10.6 (4.7, 24.1)
**Final Multivariable model**	5,360	2.8 (2.2, 3.5)	1	2.1 (1.6, 2.7)	9.6 (6.3, 14.5)	9.8 (4.3, 22.0)

a Kidney disease was classified based on estimated glomerular filtration rates (normal > = 90, mild 60–89, moderate 30–59, severe <30 mL/min/1.73 m^2^). Gout was defined as self-reported physician/provider diagnosis. See methods section for details. Hyperuricemia was defined as serum urate >7.0 mg/dL (>416 micromoles/L). Final multivariable models adjusted for age, gender, ethnicity, body mass index, hypertension status, diabetes status, use of antihypertensive medications, log-transformed blood lead level and hyperlipidemia status. Prevalence rates given as percentage (95% confidence interval).

b One standard deviation of eGFR was 27.6 mL/min/1.73 m^2^.

c Exact confidence interval (1.03, 3.41).

d Exact confidence interval (1.02, 3.20).

**Table 7:** Results of ordinary least squared regression analyses of the relationship between serum urate and eFGR.^a^

	Number of observations	Increase in serum urate (mg/dL) for eachstandard deviation decrease in eGFR(95% confidence interval)^b^	Model fit (R^2^)
**Unadjusted**	5,589	0.05 (0.45, 0.55)	7%
**Adjusted for age, sex, gender and race**	5,589	0.69 (0.62, 0.77)	30%
**Final Multivariable model**	5,363	0.64 (0.56, 0.72)	41%

a Gout was defined as self-reported physician/provider diagnosis. See methods section for details. Hyperuricemia was defined as serum urate>7.0 mg/dL (>416 micromoles/L). Glomerular filtration rate was estimated per CKD-EPI equation. Final multivariable model adjusted for age, gender, ethnicity, body mass index, hypertension status, diabetes status, use of antihypertensive medications, log-transformed blood lead level and hyperlipidemia status.

b One standard deviation of eGFR was 27.6 mL/min/1.73 m^2^.

## Discussion

Renal tubules have long been thought to be the key regulatory site for urate excretion; the role of the glomeruli has been thought to be minor, except in advanced kidney disease. [7], [37] The present study suggests that glomerular function may play a larger role in regulating serum urate than previously thought.

Although numerous studies have shown a high prevalence of renal impairment among those with gout, there have been surprisingly few published data on the prevalence of gout among those with renal impairment. The present study showed that those in the severe renal impairment category had a 6-fold increase in prevalence of gout and a 20-fold increase in the prevalence of hyperuricemia. In all analyses the most marked increase in the prevalence of gout and hyperuricemia was documented among those with moderate or severe renal impairment (eGFR was below 60 mg/min/1.73 m^2^). The odds ratio of gout among those with mild renal impairment was more modest. This could be a reflection of misclassification due to an inaccurate definition of mild renal impairment category, engendered by reliance on a single creatinine measurement. Another possibility is that this reflects the compensatory increases in renal and fecal excretion of urate in response to decreased glomerular function. [7].

The prevalence rates of gout among African Americans were the highest, and were the lowest among Hispanics as observed in previous studies; [26] however these differences were not statistically insignificant. African Americans are at greater risk for renal impairment by virtue of their metabolic risk factors, and their gout prevalence rates were higher than those of Whites. [38] Although Hispanics have been reported to have a greater risk for incident renal impairment and worse risk for progression, the opposite was observed in terms of overall gout prevalence and gout prevalence as a function of severity of renal impairment. This result must be interpreted cautiously, as the extent of bias engendered by a case definition of gout that presupposes physician/provider access, volunteer bias and adequacy of Spanish language translations can only be addressed by prospective follow up studies.

The findings of the present study must be considered in the context of the limitations of the NHANES study. The cross sectional design precludes causal inferences, a major methodological drawback of the present analyses. Nevertheless, Mendelian randomization analyses show that polymorphisms of urate transport increased the risk for hyperuricemia and gout but not renal impairment. [39] Significant misclassification errors were possible with respect to subjects in the mild renal impairment category even though the renal impairment-EPI equations are expected to be more precise and accurate than older methods. Such a misclassification of individuals having no renal impairment as having mild renal impairment could have resulted in an underestimate of the true prevalence of gout in this category. Furthermore, direct comparison with other studies requires caution; differences in methods used to assess gout and renal dysfunction may explain some of the observed differences in the prevalence of gout.

Within the population with gout the prevalence rate of renal impairment was higher than the ∼40% previously reported from managed care data and gout registries. [17], [18] Urate control in gout is poor among those without renal impairment and is worse among those with renal impairment. [18] None of the available urate lowering therapies has been designated safe and effective for patients with renal impairment. Indeed, presence of renal impairment reduces efficacy and increases the risk of serious adverse events with allopurinol, for example. [40], [41] Nevertheless, early studies suggest urate control may improve physiological parameters of glomerular function, an observation that merits further investigation an avenue of research that holds much promise. [42], [43], [44].
